# Supplementary material for: A Molecular Dynamics Study of Monomer Melt Properties of Cyanate Ester Monomer Melt Properties
Source: Polymers (Basel). 2022 Mar 17;14(6):1219. doi: 10.3390/polym14061219 (PMC8951156; doi:10.3390/polym14061219)
Supplement: Supplementary file 1 [file polymers-14-01219-s001.zip › polymers-1613674-supplementary.pdf]

Supplementary Material

A molecular dynamics study of cyanate ester monomer melt properties

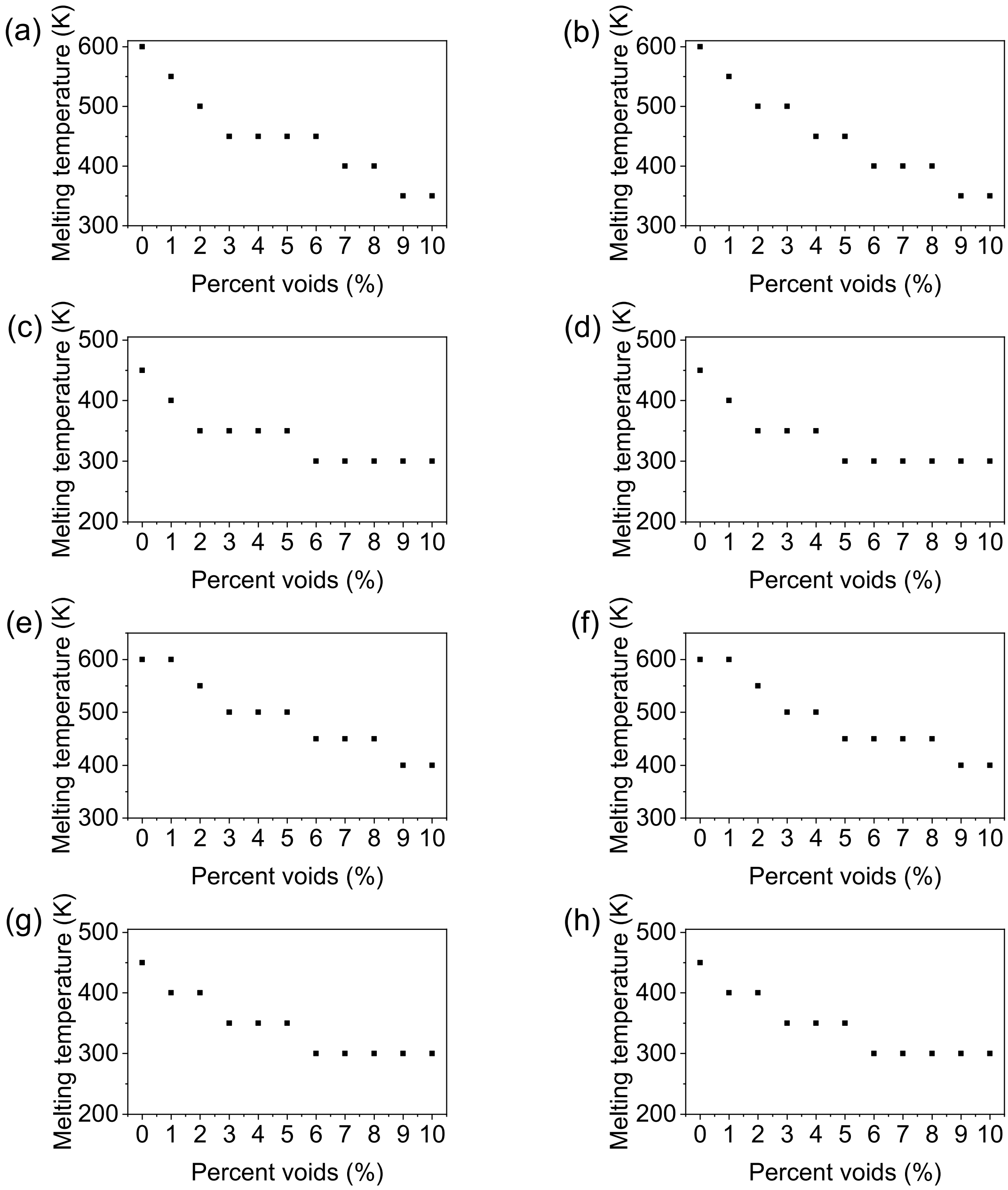

Figure S1. The effect of ensemble size on the calculated melting temperature for: (**a**) BADCy (256 molecules), (**b**) BADCy (400 molecules), (**c**) LECy (256 molecules), (**d**) LECy (400 molecules), (**e**) SiCy-3 (256 molecules), (**f**) SiCy-3 (400 molecules), (**g**) SiMCy (256 molecules), and (**h**) SiMCy (400 molecules).

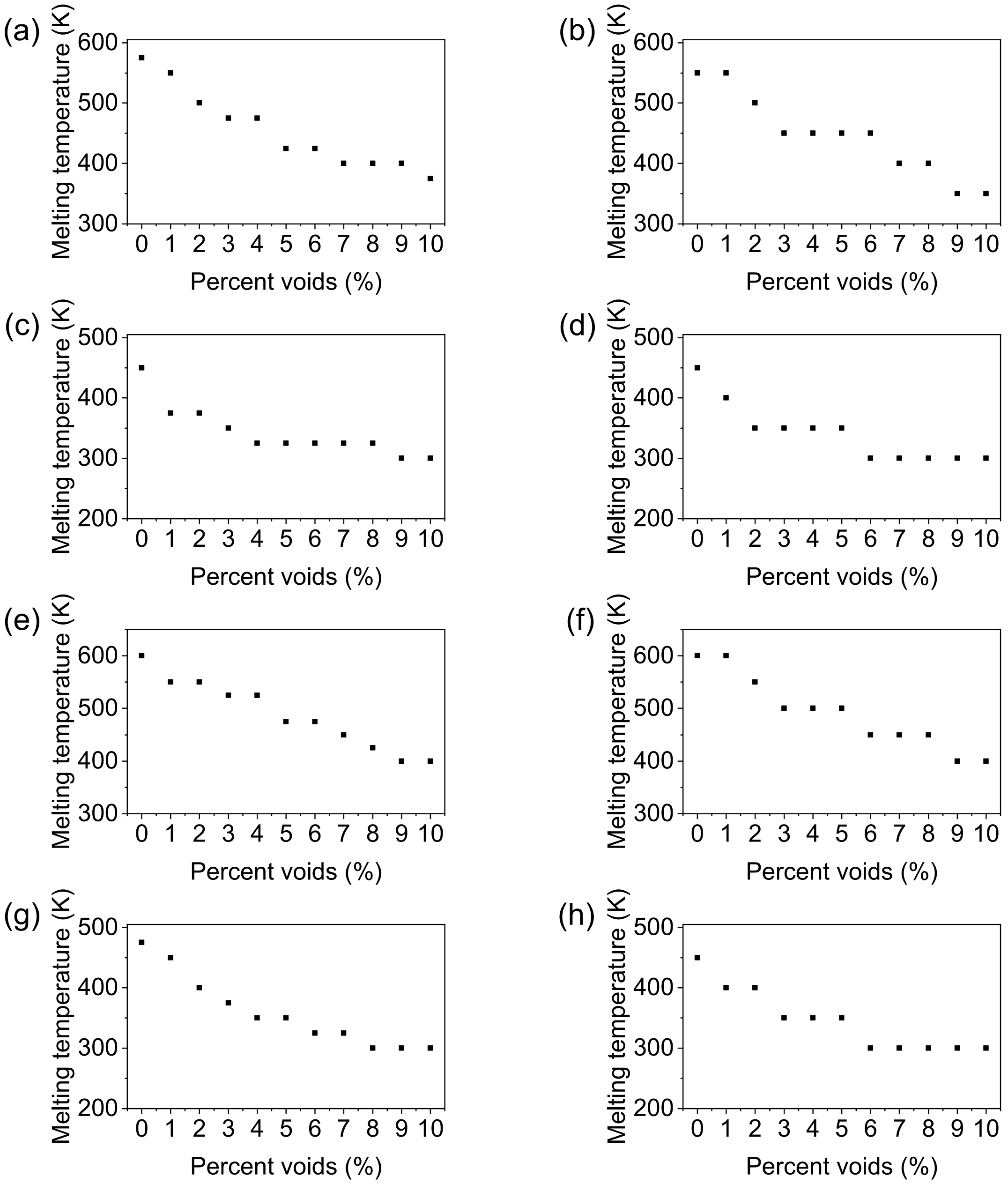

Figure S2. The effect of heating rate on the calculated melting temperature for: (**a**) BADCy (50 K/ 20 ns), (**b**) BADCy (25 K/ 20 ns), (**c**) LECy (50 K/ 20 ns), (**d**) LECy (25 K/ 20 ns), (**e**) SiCy-3 (50 K/ 20 ns), (**f**) SiCy-3 (25 K/ 20 ns), (**g**) SiMCy (50 K/ 20 ns), and (**h**) SiMCy (25 K/ 20 ns).

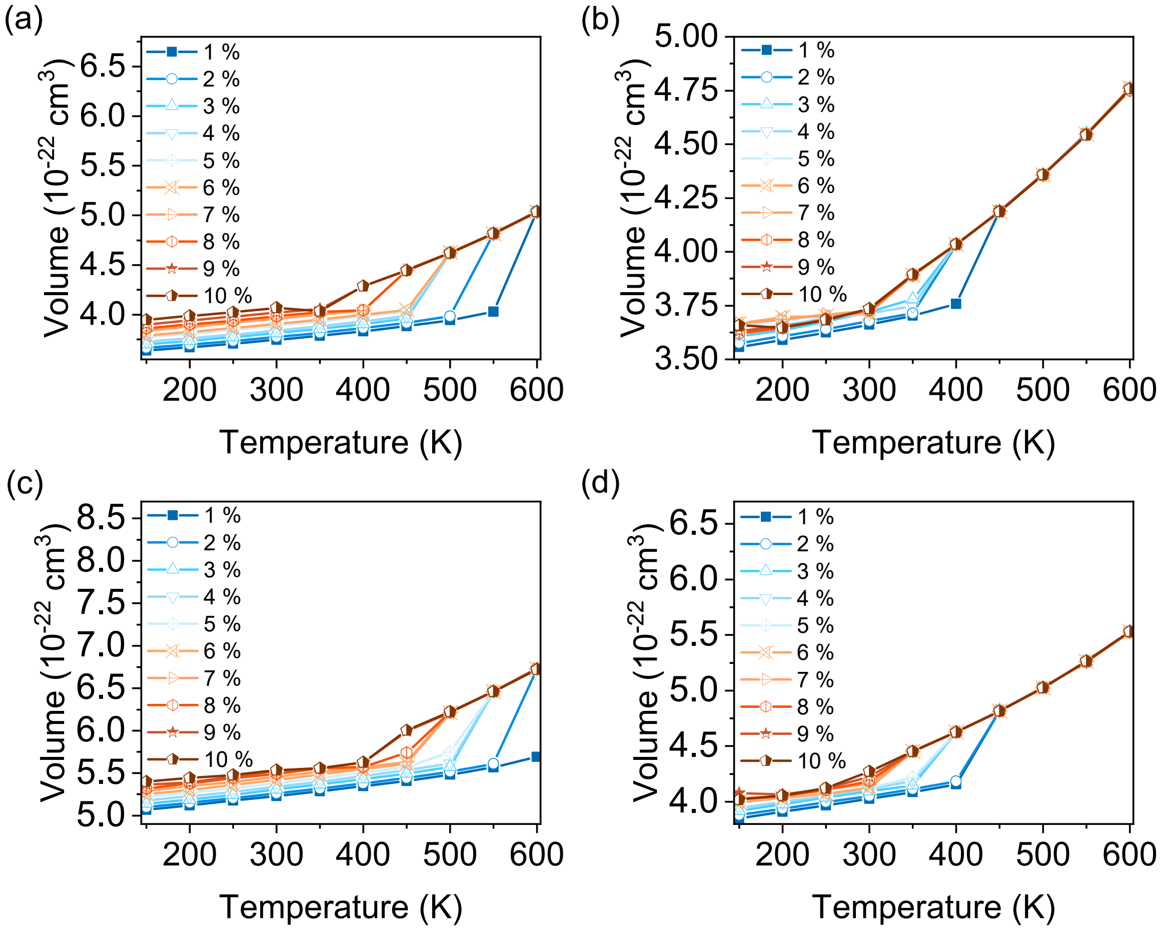

Figure S3. The evolution of volume as a function of temperature starting at T = 150 K for (**a**) BADCy, (**b**) LECy, (**c**) SiCy-3, and (**d**) SiMCy with 1 to 10% voids.

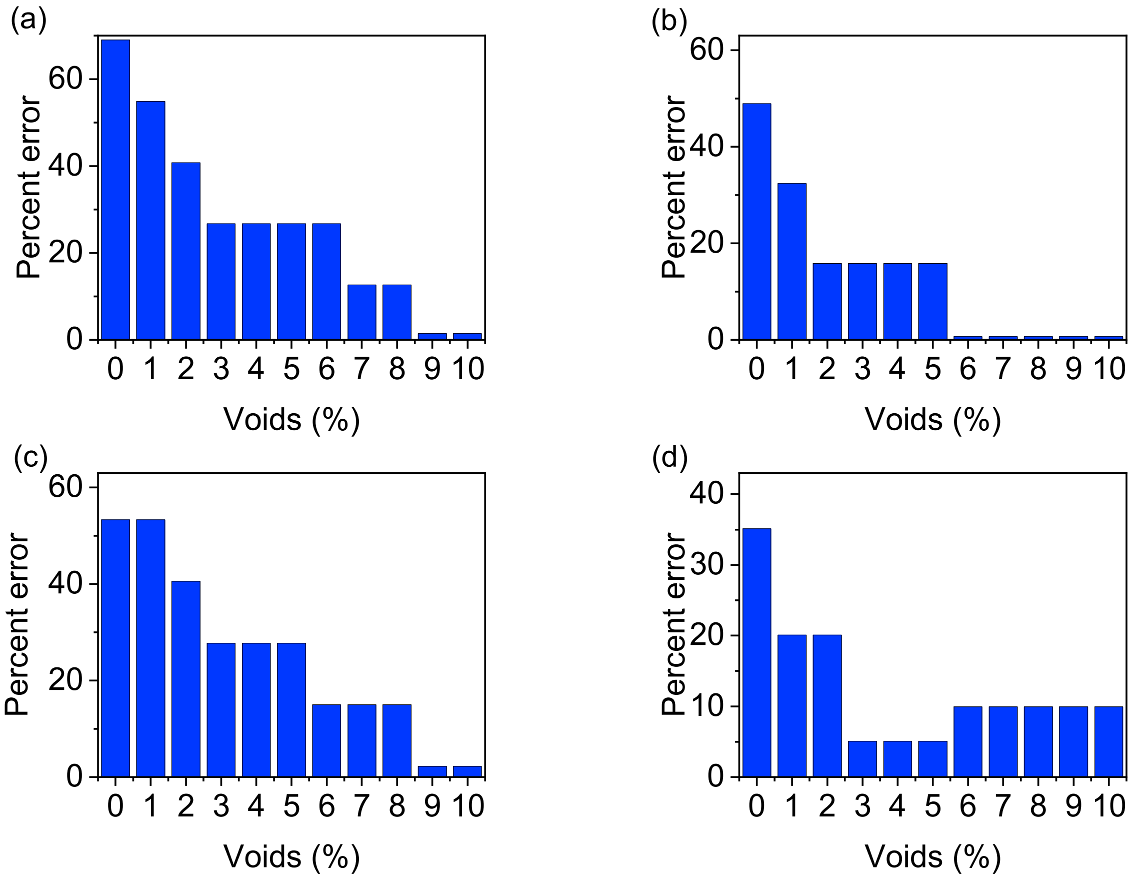

Figure S4. Percent error of the calculated melting temperature, compared to the experimental values, for simulations with 0 to 10% voids (in % of the total monomers removed from simulation cell) for: (**a**) BADCy, (**b**) LECy, (**c**) SiCy-3, and (**d**) SiMCy.

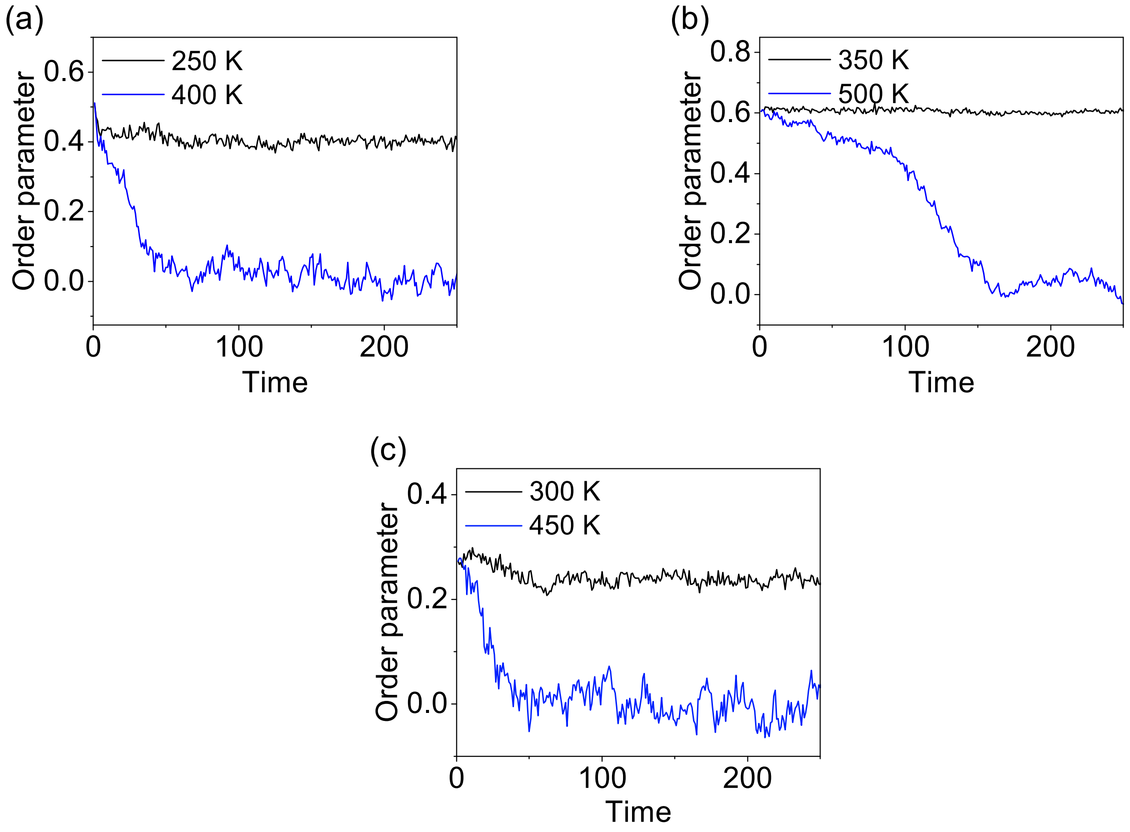

Figure S5. The orientational order parameter for monomers: (**a**) LECy, (**b**) SiCy-3, and (**c**) SiMCy 50 K below the calculated melting temperature (black line) and 50 K above the calculated melting temperature (blue line).

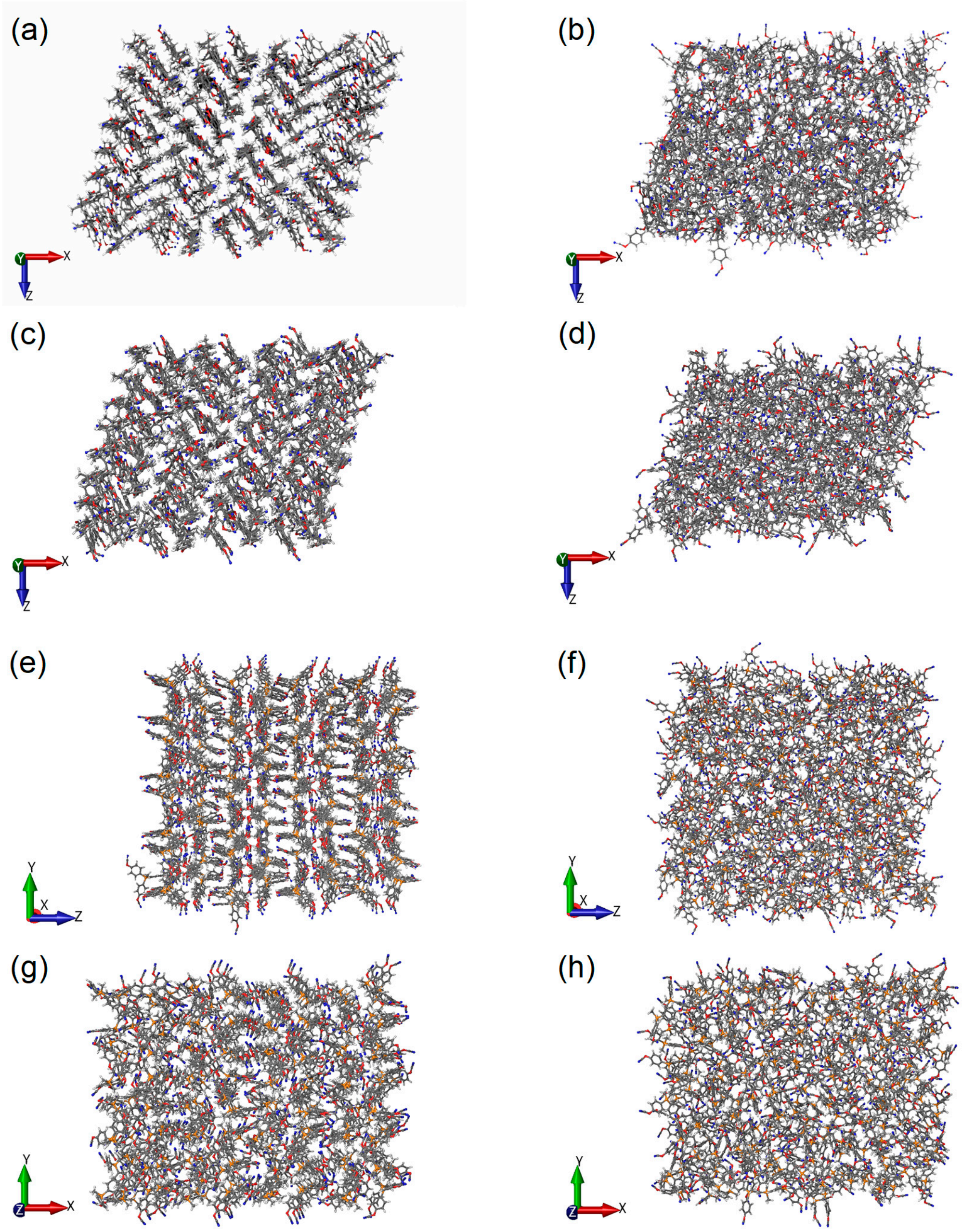

Figure S6. Snapshots of the monomers at different stages of the melting process with 9% voids for the following monomers: (**a**) BADCy (300 K), (**b**) BADCy (450 K), (**c**) LECy (250 K), (**d**) LECy (400 K), (**e**) SiCy-3 (350 K), (**f**) SiCy-3 (500 K), (**g**) SiMCy (250 K), and (**h**) SiMCy (400 K).

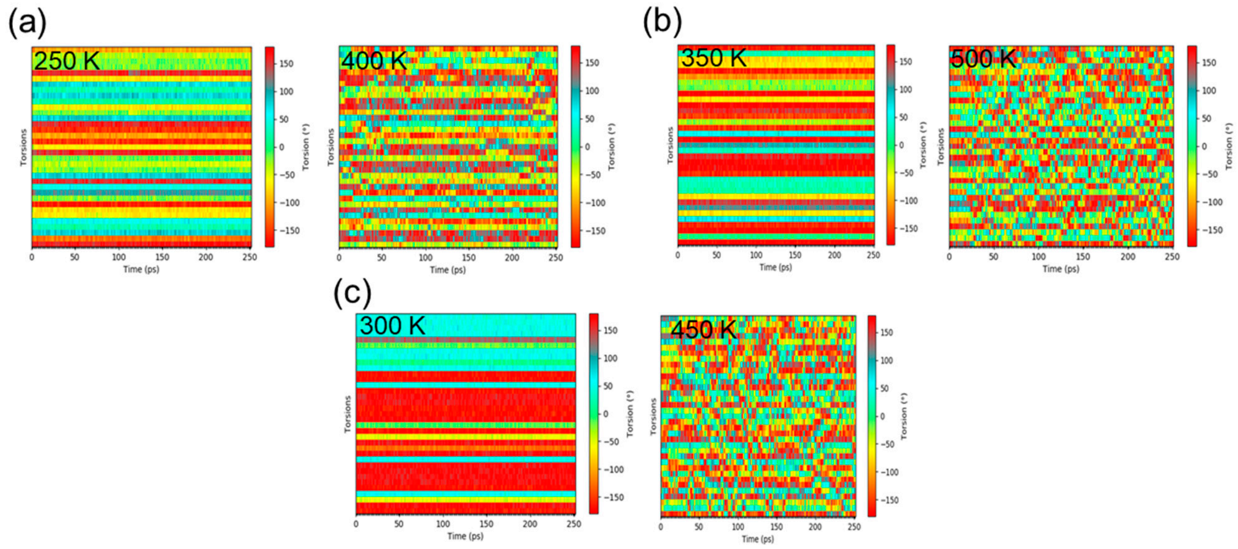

Figure S7. Display of angles for each individual torsion as a bar plot over the simulation time range with colors denoting the size of the angle for: (**a**) LECy, (**b**) SiCy-3, (**c**) SiMCy.
